# Isoprene improves photochemical efficiency and enhances heat dissipation in plants at physiological temperatures

**DOI:** 10.1093/jxb/eru033

**Published:** 2014-03-25

**Authors:** Susanna Pollastri, Tsonko Tsonev, Francesco Loreto

**Affiliations:** ^1^The National Research Council of Italy (CNR), Department of Biology, Agriculture and Food Sciences, Institute for Plant Protection, Via Madonna del Piano 10, 50019 Sesto Fiorentino (Florence), Italy; ^2^Bulgarian Academy of Sciences, Institute of Plant Physiology and Genetics, Acad. G. Bonchev Str., Bl. 21, 1113 Sofia, Bulgaria; ^3^The National Research Council of Italy (CNR), Department of Biology, Agriculture and Food Sciences, Piazzale Aldo Moro 7, 00185 Rome, Italy

**Keywords:** Chloroplast functionality, climate change, fluorescence quenching, high temperature, isoprene, photosynthesis, stress physiology.

## Abstract

Isoprene-emitting plants are better protected against thermal and oxidative stresses. Isoprene may strengthen membranes avoiding their denaturation and may quench reactive oxygen and nitrogen species, achieving a similar protective effect. The physiological role of isoprene in unstressed plants, up to now, is not understood. It is shown here, by monitoring the non-photochemical quenching (NPQ) of chlorophyll fluorescence of leaves with chemically or genetically altered isoprene biosynthesis, that chloroplasts of isoprene-emitting leaves dissipate less energy as heat than chloroplasts of non-emitting leaves, when exposed to physiologically high temperatures (28–37 °C) that do not impair the photosynthetic apparatus. The effect was especially remarkable at foliar temperatures between 30 °C and 35 °C, at which isoprene emission is maximized and NPQ is quenched by about 20%. Isoprene may also allow better stability of photosynthetic membranes and a more efficient electron transfer through PSII at physiological temperatures, explaining most of the NPQ reduction and the slightly higher photochemical quenching that was also observed in isoprene-emitting leaves. The possibility that isoprene emission helps in removing thermal energy at the thylakoid level is also put forward, although such an effect was calculated to be minimal. These experiments expand current evidence that isoprene is an important trait against thermal and oxidative stresses and also explains why plants invest resources in isoprene under unstressed conditions. By improving PSII efficiency and reducing the need for heat dissipation in photosynthetic membranes, isoprene emitters are best fitted to physiologically high temperatures and will have an evolutionary advantage when adapting to a warming climate.

## Summary

At physiological temperatures, chloroplasts of isoprene-emitting leaves dissipate less energy as heat than chloroplasts of non-emitting leaves, determining a more efficient electron transfer through PSII.

## Introduction

Isoprene (C_5_H_8_) is a volatile molecule synthesized in the chloroplasts of many plant species through the photosynthesis-dependent 2-C-methyl-d-erythritol 4-phosphate (MEP) pathway (Lichtenthaler *et al.*, 1997). Isoprene biosynthesis is costly, both in terms of carbon and of energy; 0.5–2% of photosynthetic carbon is re-emitted in the atmosphere as isoprene and this percentage increases dramatically in stressed leaves (Sharkey and Yeh, 2001). The reward for plants affording the high cost of isoprene biosynthesis has been identified; isoprene emitters are better protected against thermal and oxidative stress, as their membranes are strengthened by isoprene lipophylic properties, and undergo denaturation at higher temperatures or higher oxidant exposure level (Sharkey and Singsaas, 1995; Loreto and Schnitzler, 2010). Isoprene-conjugated double bonds also allow this molecule to react with many reactive chemical species; *in planta*, isoprene scavenges dangerous reactive oxygen and nitrogen species (Vickers *et al.*, 2009*a*); in the atmosphere, isoprene reacts with ubiquitous OH and with NOx, thus regulating the oxidizing capacity of the atmosphere, enhancing the life-time of less reactive compounds, and bringing episodes of ozone formation (Di Carlo *et al.*, 2004).

Isoprene protection of photosynthetic membranes against thermal stresses has been postulated theoretically (Siwko *et al.*, 2007) and recently demonstrated by three biophysical measurements (Velikova *et al.*, 2011). However, whether isoprene emission also produces positive effects at physiologically high temperatures that do not permanently impair membrane structure and function (e.g. when photosynthesis is still largely unaffected, except for the physiological inhibition due to the enhanced competition of photorespiration for Rubisco at high temperatures) is currently unknown. Recent work has highlighted lower non-photochemical quenching (NPQ) of chlorophyll fluorescence in isoprene-emitting poplars with respect to non-emitting transgenics, implying that the electron transfer flows better, and there is less need for heat dissipation, in isoprene emitters. However, this was only clearly demonstrated in leaves exposed to repeated cycles of heat stress (Behnke *et al.*, 2007; Way *et al.*, 2012). It may be hypothesized that isoprene, by making the membranes more resistant to thermal stress, would also make them operating better under physiologically high temperatures.

## Materials and methods

### Plant material

Plants of *Populus nigra, Nicotiana tabacum,* and *Arabidopsis thaliana* were used. *Populus nigra* (black poplar) is a plant species that naturally emits isoprene (Guidolotti *et al.*, 2011). Wild-type *Nicotiana* (tobacco) and *Arabidopsis* do not emit isoprene, but transgenic, isoprene-emitting lines of *Nicotiana* (Vickers *et al.*, 2009*b*) and *Arabidopsis* (Sharkey *et al.*, 2005) were used to perform this experiment.

Two-year-old *Populus* were grown outdoors in 20 l pots with commercial substrate. *Nicotiana* and *Arabidopsis* plants were grown in 2.0 l and 0.5 l pots, respectively, with commercial soil substrates, in two different climatized phytotrons under the following conditions: for *Nicotiana*, ambient temperature: 26/24 °C (day/night) and photosynthetic photon flux density (PPFD): 800±100 μmol m^–2^ s^–1^. For *Arabidopsis*: ambient temperature: 21/20 °C (day/night) and PPFD: 300±100 μmol m^–2^ s^–1^.

All the plants used to perform this experiment were maintained under well-watered and well-fertilized conditions. Irrigation was performed daily, while fertilizers were added weekly to the water, using a full-strength Hoagland solution.

### Measurements

Measurements were carried out on single leaves of poplar, cut under water and transferred in 2ml Eppendorf vials filled with water. Part of the leaf was enclosed in a 2cm^2^ gas-exchange cuvette, and exposed to simultaneous gas-exchange and fluorescence measurements using a Li-Cor 6400-60-XT gas-exchange system (Li-Cor Lincoln, Nebraska, USA). Gas-exchange measurements allowed direct calculations of photosynthesis and stomatal conductance using the instrumental software. Isoprene was measured using a gas chromatograph (BTX Analyser GC 855, Syntech Spectras, Groningen, The Netherlands) that collected on-line 100ml of air at the cuvette output every 6min. The maximal quantum yield of PSII was determined after dark-adapting the leaf for 30min. The leaf was then exposed to 1000 µmol photons m^–2^ s^–1^ of PPFD, and gas-exchange and chlorophyll fluorescence parameters were determined on leaves after reaching steady-state conditions. The NPQ, which was calculated as *F*_m_/$F_{\text{m}}^{�}$–1 (where *F*_m_ and $F_{\text{m}}^{�}$ are the maximal fluorescence in dark- and light-adapted leaves, respectively) estimated the rate constant for heat loss from PSII. The photochemical fluorescence quenching (qP), and the PSII quantum yield (Φ_PSII_) were calculated as qP=($F_{\text{m}}^{�}$ −*F*)/($F_{\text{m}}^{�}$−$F_{\text{o}}^{�}$), and Φ_PSII_=($F_{\text{m}}^{�}$–*F*)/$F_{\text{m}}^{�}$, respectively, where $F_{\text{o}}^{�}$ is the minimum fluorescence, and *F* is the steady-state fluorescence in light-adapted leaves. Updated information on chlorophyll fluorescence parameters and their physiological meaning can be retrieved from Baker (2008). The sequence of measurements was repeated 30min after inhibiting leaf isoprene emission by feeding 20 µM fosmidomycin to the water in the vial (Loreto and Velikova, 2001). Fosmidomycin is a competitive inhibitor which completely inhibits isoprene when taken up through the transpiration stream activated by open stomata (Loreto and Velikova, 2001). In our case, with stomatal conductances between 0.21 and 0.25mol m^–2^ s^–1^, isoprene emission was inhibited within 30min with no effect on other physiological parameters. Side-effects of fosmidomycin are reported when feeding occurs for a longer time-course under saturating light intensity or when higher concentrations are used and the metabolism of other isoprenoids is also impaired (Possell *et al.*, 2010). Measurements on detached leaves before and after feeding fosmidomycin were made at leaf temperatures of 28, 30, 32, 35, and 37 °C, i.e. up to temperatures that may lead to a down-regulation of photosynthesis but do not permanently impair photochemical efficiency and overall photosynthetic metabolism, as demonstrated by basal fluorescence stability (Sharkey, 1996). Care was taken to compare leaves with similar photosynthetic and stomatal conductance rates, to rule out changes of NPQ due to uneven photochemistry and different latent heat release through the stomata. Each leaf was only measured at one temperature. Measurements at each temperature were repeated on six different leaves. In a different experiment, in order to reconstitute the isoprene pools in the chloroplasts of those leaves whose endogenous isoprene emission had previously been inhibited by fosmidomycin, 2–3 ppm of gaseous isoprene were supplied as in Loreto *et al.* (1998), and physiological parameters were recorded before and after isoprene inhibition, at 32 °C.

Measurements were repeated on non-emitting and on transformed, isoprene-emitting *Arabidopsis* and *Nicotiana* plants. *Nicotiana* leaves were exposed to the same temperature conditions used with *Populus*, while *Arabidopsis* was only exposed to 30 °C. With both *Arabidopsis* and *Nicotiana* plants, the electron transport parameters from non-emitting wild types and from isoprene-emitting, genetically engineered plants were compared on selected leaves that showed similar photosynthesis and stomatal conductance, in order to avoid interfering effects of physiological differences on NPQ determination and leaf temperature, as shown above for *Populus*.

### Data presentation and statistics

**Fig. 1.**
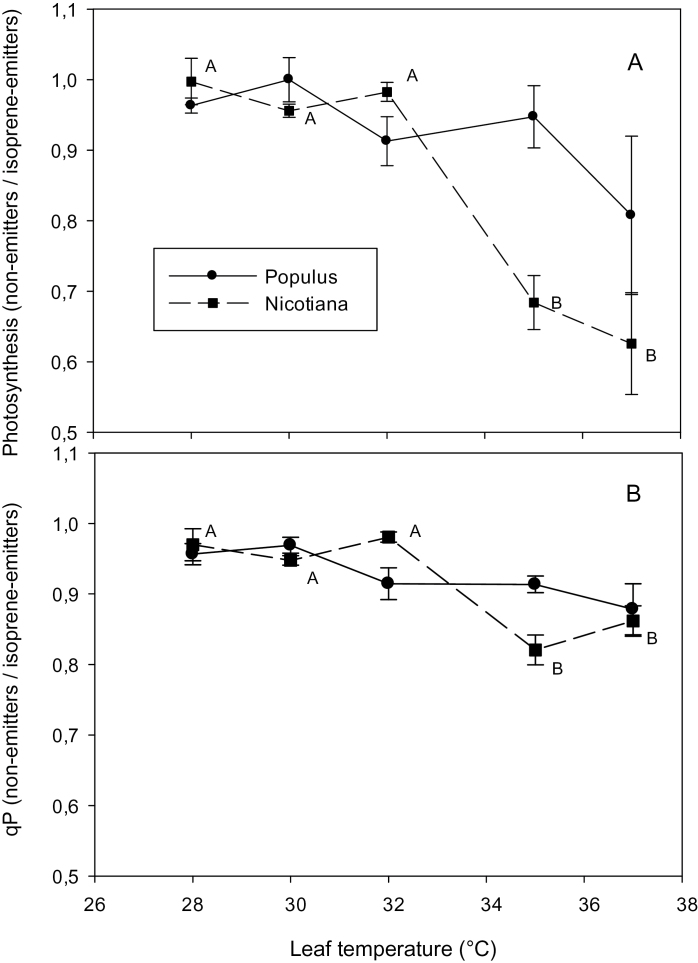
(A) The ratio of photosynthesis and (B) photochemical quenching of chlorophyll fluorescence (qP) in non-emitting/isoprene-emitting leaves at rising temperatures, in *Populus* (black circles) and *Nicotiana* (black squares). Each data-point shows the mean ±standard error of six measurements made on different leaves. Different letters identify values significantly different over the temperature range on *Nicotiana* plants, according to Tukey’s test (*P* <0.05). None of the measured values of photosynthesis and *qP* were statistically different in *Populus*, following the same statistical test.

**Fig. 2.**
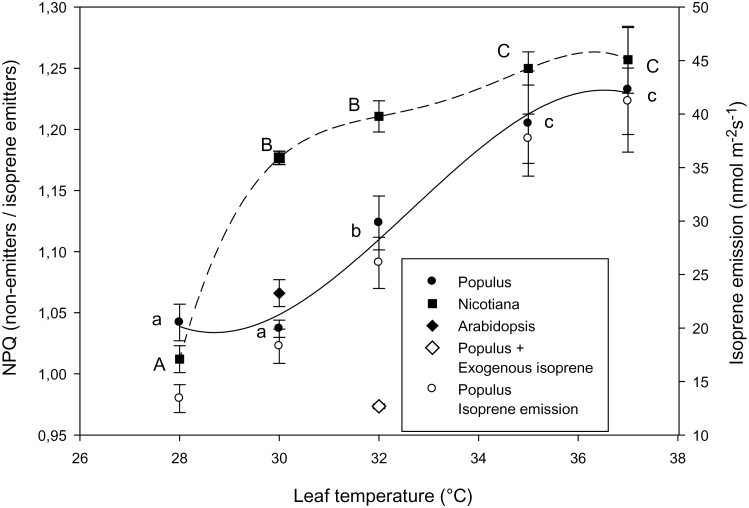
The ratio of non-photochemical quenching (NPQ) of chlorophyll fluorescence in non-emitting/isoprene-emitting leaves, at rising temperatures, in *Populus* (black circles) and *Nicotiana* (black squares). The same measurements were repeated at 30 °C in *Arabidopsis* (black diamond) and at 32 °C in *Populus* leaves in which endogenous isoprene had been inhibited by fosmidomycin, and then restored by feeding exogenous isoprene (white diamond). The third order best-fits describe a sigmoidal relationship in *Populus* (*r* ^2^=0.98), with an exponential increase in NPQ in isoprene-inhibited *Populus* leaves mirroring the temperature response of the emission in leaves that naturally emit isoprene (white circles). A similar temperature-dependency was observed in *Nicotiana* (*r* ^2^=0.96) although a large increase of NPQ had already been observed at 30 °C. Each data-point shows the mean ±standard error of six measurements made on different leaves. NPQ data for plants that were sampled over the entire temperature range were subjected to ANOVA, followed by Tukey’s test. Means that are significantly different (*P* <0.05) are shown with different letters (upper case, *Nicotiana*; lower case, *Populus*).

On each collected data-point, and for each parameter, measurements were repeated on six different leaves of different plants. Data are shown as ratios of values in non-emitting and isoprene-emitting leaves, as this helped to normalize the results (Figs 1, 2). The means of ratios ±standard errors were statistically separated over the temperature range by ANOVA, followed by Tukey’s test (*P* <0.05). Third-order polynomial best-fits were used to describe the sigmoidal increase of the ratio of NPQ in non-emitting/isoprene-emitting plants at increasing temperatures in poplar and tobacco.

Absolute values of measured parameters are reported in Supplementary Table S1 at *JXB* online. Means ±standard errors, within each temperature treatment, were subjected to Student’s *t* test (*P <0.05 and **P <0.01) in order to identify the effect of isoprene. All statistical analyses were conducted using the SigmaPlot 12.0 software (SPSS; http://www.spss.com/).

## Results

To test experimentally whether isoprene allows the photochemistry of photosynthesis to work more efficiently under unstressed conditions, the non-photochemical quenching of chlorophyll fluorescence in leaves that emit or do not emit isoprene have been measured under physiological temperatures. Chlorophyll fluorescence is a sensitive indicator of photosystem II (PSII) photochemistry (Baker, 2008). Chlorophyll fluorescence is quenched *in vivo* by various processes. The qP mirrors the electron flow through the linear electron transport chain. The NPQ specifically estimates the amount of energy that is not used by the linear electron transport for primary metabolism, being instead dissipated as heat into PSII (Baker, 2008). Since PSII is assembled into the chloroplast membranes, NPQ may also indicate heat dissipation at the specific location where isoprene is also formed (Loreto and Schnitzler, 2010).

The photosynthetic rate, the two fluorescence quenchings, and the quantum yield of PSII, were measured in fully developed leaves of poplar which were emitting high rates of isoprene (18.3±1.6 nmol m^–2^ s^–1^ at 30 °C and 1000 µmol m^–2^ s^–1^ PPFD), and also in the same leaves after a 30-min feeding of fosmidomycin selectively to inhibit isoprene biosynthesis without affecting primary metabolism (photosynthesis or photosynthetic electron transport rate) and any other process that is known to feed back on chlorophyll fluorescence (e.g. state-transition or photoinhibition) (Loreto and Velikova, 2001).

In *Populus*, photosynthesis and the photochemical quenching of chlorophyll fluorescence were not statistically different in isoprene-emitting and isoprene-inhibited leaves at temperatures as high as 35 °C, but dropped more (yet not statistically significantly) in isoprene-inhibited than in isoprene-emitting leaves, when the temperature reached 37 °C (Fig. 1; see Supplementary Table S1 at *JXB* online). At each temperature, a reduction in Φ_PSII_ was observed when isoprene emission was suppressed, but this reduction was not statistically significant at any temperature of the range (see Supplementary Table S1 at *JXB* online).

The NPQ increased after inhibiting isoprene emission in poplar leaves at all the temperatures tested (Fig. 2). A sigmoidal response to temperature was observed in poplar, the NPQ of non-emitting leaves increasing exponentially and statistically significantly at foliar temperatures between 30 °C and 35 °C, a temperature range over which isoprene emission also increases exponentially (Niinemets *et al.*, 2004). Indeed, isoprene emission Q_10_ (25–35 °C)=2.8 in our experiment (Fig. 2). The NPQ only increased marginally at temperatures higher than 35 °C, at which temperature isoprene emission also started to level off and photosynthesis and qP started dropping in non-emitting leaves. The NPQ of isoprene-emitting leaves was not restored once isoprene-inhibited leaves were fumigated with gaseous isoprene (Fig. 2, open diamond, at 32 °C).

The experiment was repeated with plants of *Arabidopsis* and *Nicotiana* that have been engineered to emit isoprene (Sharkey *et al.*, 2005; Vickers *et al.*, 2009*b*). Isoprene emissions at 30 °C were 5.7±0.8 nmol m^–2^ s^–1^ in *Nicotiana* and 1.9±0.4 nmol m^–2^ s^–1^ in *Arabidopsis*. Photosynthesis and the photochemical quenching of fluorescence were slightly lower in non-emitting than in isoprene-emitting tobacco plants, but the difference was below 10% and statistically significant only at the highest temperatures, when the two parameters dropped considerably more in non-emitters than in isoprene-emitting plants (Fig. 1). The Φ_PSII_ was also lower in non-emitting than in emitting plants, but this difference was not statistically significant at any temperature of the range (see Supplementary Table S1 at *JXB* online). A significantly lower NPQ was also observed in isoprene-emitting leaves of *Nicotiana* and *Arabidopsis*, compared with the wild types that are unable to synthesize isoprene, and the effect was already especially strong and statistically significant in tobacco leaves at temperatures as high as 30 °C (Fig. 2).

## Discussion

The functional roles attributed to isoprene so far are concentrated on the protection offered by this volatile molecule against heat and ozone stress (Vickers *et al.*, 2009*a*), but this does not explain why isoprene is emitted at high rates when the photosynthetic apparatus is undamaged by stresses. In the absence of a clear identification of an alleged functional mechanism under unstressful conditions, the emission of isoprene has often been considered as a ‘safety valve’ to remove carbon or energy when plants cannot (or do not need to) invest it into essential isoprenoids, namely carotenoids and other products of the MEP pathway with well-established structural or protective roles (Owen and Penuelas, 2005).

Our finding expands the current notion that isoprene protects photosynthetic membranes (Sharkey and Singsaas, 1995; Velikova *et al.*, 2011), and also reveals an advantage of isoprene-emitting plants under physiological conditions in which photosynthesis is not inhibited by thermal stress, though it may be reduced by enhanced competition of photorespiration for Rubisco (Labate *et al.*, 1990) and by enhanced mitochondrial respiration (Atkin *et al.*, 2005; Fares *et al.*, 2011). Further evidence is also supplied that the emission of isoprene has been evolutionarily maximized at high temperatures to reduce the pressure on membranes and to quench the need to dissipate energy non-radiatively (Sharkey and Yeh, 2001).

Specifically, our experiments revealed that the non-photochemical quenching (NPQ) of fluorescence is reduced in the three species that were tested when they are genetically programmed to emit isoprene with respect to when isoprene is not produced. A larger increase of NPQ was found in plants undergoing one or more cycles of heat stress and in which isoprene emission was suppressed, compared with isoprene-emitters (Behnke *et al.*, 2007; Way *et al.*, 2011), but it was found here that this is also true in unstressed plants. In poplar, NPQ reduction was dependent on the internal concentration of isoprene (Fig. 2), with a 20% NPQ reduction at 34–37 °C foliar temperatures, levelling off coincidentally with maximal isoprene production and emission. In tobacco, the NPQ increased significantly more in non-emitters than in emitters when the temperature was set at 30 °C or higher temperatures.

A lower temperature-dependent down-regulation of photosynthesis could explain the reduced need for energy dissipation in isoprene-emitting plants. No damage to photosynthesis was observed after exposure to the physiologically high temperatures reproduced in our experiments, as the basal fluorescence (Sharkey, 1996) was similar in all leaves (not shown). There was no significant change in photosynthesis and photochemical quenching of fluorescence (qP) in leaves monitored at up to 35 °C, whereas a large increase of NPQ occurred at lower temperatures, both in poplar and tobacco. Thus, there is no direct evidence that the increasing need to dissipate energy as NPQ is related to significantly lower photosynthesis when isoprene is absent. However, it is noted from Fig. 2 and from Supplementary Table S1 at *JXB* online that photosynthesis, qP, and Φ_PSII_, in the absence of isoprene: (i) were only slightly, but constantly lower than in isoprene-emitting poplar and tobacco plants when monitored at the lower temperatures of our range (28–32 °C) and (ii) were largely, though not permanently (and in the case of Φ_PSII_ statistically non-significantly) reduced when temperatures exceeded 32 °C in tobacco and 35 °C in poplar. It is therefore surmised that isoprene allows the photosynthetic machinery to work closer to optimal conditions even at physiological temperatures, maintaining optimal membrane viscosity and functioning of lipo-proteic structures, as already theoretically hypothesized (Siwko *et al.*, 2007) and experimentally observed under more extreme temperatures (Singsaas *et al.*, 1997; Velikova *et al.*, 2011). Maintenance of chloroplast stability under heat pressure would also avoid the formation of reactive oxygen species because of inefficient photosynthetic electron transport, as also seen in isoprene-emitting leaves (Loreto and Velikova, 2001; Velikova *et al.*, 2012).

However, membrane strengthening would be better achieved by non-volatile molecules (e.g. carotenoids), so isoprene might also thermally protect leaves by a different mechanism. Leaves cool down by the evaporation of water through stomatal apertures. This well-known process (Curtis, 1936) allows foliar thermal regulation over a large span of temperatures, especially when leaves directly intercept infrared radiation on sunny days (Jones, 1999). Similarly, isoprene evaporation could turn the chloroplasts cooler. Isoprene is emitted at rates 10^–6^ lower than water, thus having a markedly lower sensible heat-dissipation effect. However, isoprene boils at only 34 °C and is virtually completely volatilized once formed, therefore instantaneously dissipating sensible heat; consistently, no permanent foliar pools of isoprene have been reported (Loreto and Schnitzler, 2010). Moreover, water vapour is released from the intercellular spaces, whereas isoprene is produced and emitted inside chloroplasts, where it could specifically exert its cooling effect. NPQ was not restored by fumigating poplar leaves, in which endogenous emission of isoprene had been chemically inhibited, with gaseous isoprene which supports the idea that the volatilization of isoprene may regulate the heat-dissipation properties of chloroplasts. It may be calculated from the physical properties of isoprene that the latent heat energy needed to vaporize one molecule of isoprene is about 27 kJ. The concentration of isoprene inside isoprene-emitting leaves is estimated to be between 500 (Sharkey *et al.*, 1996; Velikova *et al.*, 2011) and 100 nmol m^–2^ (Loreto *et al.*, 1998), when measured at 34 °C. Considering the highest concentration, the evaporation of isoprene would remove about 13.5×10^–3^ J of latent heat. Although low in absolute terms, this is enough theoretically to reduce the temperature of the phospho-lipid bilayer of the chloroplast membranes where isoprene resides. Since the specific heat of this bilayer is 2093 J kg^–1^ °C^–1^ (Blume, 1983), and the weight of chloroplasts in a leaf is nearly 7g m^–2^ (Ivanova *et al.*, 2009), it can be estimated that, at temperatures above 34 °C, the instantaneous evaporation of isoprene reduces the temperature of the chloroplast membranes by at least 0.001 °C. A higher temperature cooling effect would, however, be possible in leaves in which the density of isoprene molecules per chloroplast is higher, or if, by crossing layers of chloroplasts before exiting the leaf, the effect is multiplied through cycles of condensation and vaporization of isoprene, or if isoprene also distributes the excess of thermal energy from chloroplasts to the other leaf (cellular and extracellular) parts, making the internal energy of the system more uniform. Strong isoprene emitters are fast-growing plants, which do not have thick layers of tissues in the mesophyll (Loreto and Schnitzler, 2010), thus the first hypothesis would be more realistic. Whether such a small thermal effect would be sufficient to make the photosynthetic cycle of isoprene-emitters more efficient under physiological and up to quasi-critical temperatures, as shown here, remains to be demonstrated. It should be also noted that, currently, no available method allows the direct measurement of chloroplast temperature *in vivo*, so the cooling effect of isoprene cannot be tested. However, if isoprene also helps to control chloroplast temperature, then heat dissipation by PSII embedded in the chloroplast membranes (NPQ) may be considered a proxy of chloroplast temperature, being NPQ enhanced when chloroplast temperature rises. The whole issue needs further testing as soon as experimentally possible.

By demonstrating that isoprene emission makes more efficient PSII photochemistry and reduces the need for heat dissipation from chloroplasts at physiologically high temperatures, the large temperature-dependent investment of carbon on isoprene by unstressed leaves becomes evolutionarily more sound. Isoprene emission is predicted to increase at a global level because current climate warming and associated stresses will positively feed back on isoprene biosynthesis (Penuelas and Staudt, 2010). The higher oxidative properties of the future atmosphere also let us predict an evolutionary advantage of isoprene emitters (Lerdau, 2007). As highlighted by this experiment, isoprene emission may become an even more important trait under current climate change, favouring plant adaptation to a rapidly warming environment.

## Supplementary Material

Supplementary Data
