# Supplementary material for: Isoprene improves photochemical efficiency and enhances heat dissipation in plants at physiological temperatures
Source: J Exp Bot. 2014 Mar 25;65(6):1565–70. doi: 10.1093/jxb/eru033 (PMC3967094; doi:10.1093/jxb/eru033)
Supplement: Supplementary Data [file supp_65_6_1565__index.html]

Isoprene improves photochemical efficiency and enhances heat dissipation in plants at physiological temperatures — Supplementary Data 

# Isoprene improves photochemical efficiency and enhances heat dissipation in plants at physiological temperatures

## Supplementary Data

Data files

**Files in this Data Supplement:**

- Supplementary Data - Supplementary Data
