# Supplementary material for: Isoprene improves photochemical efficiency and enhances heat dissipation in plants at physiological temperatures
Source: J Exp Bot. 2014 Mar 25;65(6):1565–70. doi: 10.1093/jxb/eru033 (PMC3967094; doi:10.1093/jxb/eru033)
Supplement: Supplementary Data [file supp_eru033_jexbot108365_file001.pdf]

Isoprene improves photochemical efficiency and enhances heat dissipation in plants at physiological temperatures.

Susanna Pollastri, Tsonko Tsonev, Francesco Loreto

Supplementary Table S1.

Populus nigra and *Nicotiana tabacum* values for: photosynthesis (A, µmol m^2 sec^{-1}), stomatal conductance (g_s, mol m^2 sec^{-1}), photochemical (qP) and non-photochemical (NPQ) fluorescence quenchings, and PSII quantum yield in the light (Φ_{PSII}). The values are averages of 6 independent measurements ± standard error. Data were recorded at different temperatures, in isoprene-emitters and non-emitters. All means, within each temperature treatment, were subjected to Student's t-test. Means significantly different between isoprene-emitters and non-emitters, at the same temperature, are shown with asterisks (* = P < 0.05 and ** = P < 0.01).

Species	Leaf T (°C)	Isoprene	A (µmol m^{2}sec^{-1})	g_s (mol m^2 sec^{-1})	qP	NPQ	ΦPSII
**Populus**	**28**	**emitters**	15 ± 0.1	0.21 ± 0.01	0.565 ± 0.013	2.75 ± 0.05	0.259 ± 0.011
		**non-emitters**	14.8 ± 0.12	0.22 ± 0.01	0.541 ± 0.012	2.87 ± 0.01 *	0.233 ± 0.007
	**30**	**emitters**	14.9 ± 0.37	0.23 ± 0.03	0.578 ± 0.018	2.47± 0.04	0.270 ± 0.011
		**non-emitters**	14.9 ± 0.52	0.22 ± 0.05	0.560 ± 0.017	2.57± 0.01 *	0.246 ± 0.007
	**32**	**emitters**	15 ± 0.49	0.23 ± 0.01	0.560 ± 0.015	2.31 ± 0.14	0.261 ± 0.017
		**non-emitters**	13.7 ± 0.65	0.25 ± 0.03	0.513 ± 0.020	2.83 ± 0.14 *	0.208 ± 0.016
	**35**	**emitters**	15.7 ± 1.05	0.23 ± 0.01	0.619 ± 0.021	2.32 ± 0.09	0.298 ± 0.020
		**non-emitters**	14.9 ± 1.24	0.24 ± 0.03	0.566± 0.025	2.74 ± 0.09 **	0.236 ± 0.021
	**37**	**emitters**	14.7 ± 1.28	0.24 ± 0.02	0.567 ± 0.040	2.74 ± 0.25	0.262 ± 0.031
		**non-emitters**	11.9 ± 1.67	0.25 ± 0.04	0.498 ± 0.047	3.43 ± 0.26 *	0.199 ± 0.027
**Nicotiana**	**28**	**emitters**	7.6 ± 0.7	0.21 ± 0.03	0.205 ± 0.014	2.44 ± 0.10	0.108 ± 0.009
		**non-emitters**	7.5 ± 0.8	0.20 ± 0.05	0.234 ± 0.019	2.47 ± 0.09 *	0.108 ± 0.007
	**30**	**emitters**	8.0 ± 0.7	0.22 ± 0.02	0.334 ± 0.087	2.05 ± 0.12	0.112 ± 0.073
		**non-emitters**	7.0 ± 0.5	0.21 ± 0.03	0.255 ± 0.011	2.39 ± 0.10 *	0.094 ± 0.056
	**32**	**emitters**	8.0 ± 1	0.23 ± 0.03	0.283 ± 0.021	1.86 ± 0.26	0.110± 0.066
		**non-emitters**	7.0 ± 0.6	0.22 ± 0.02	0.278 ± 0.016	2.59 ± 0.11 *	0.090 ±0.046
	**35**	**emitters**	7.9 ± 0.9	0.20 ± 0.02	0.339 ± 0.023	1.71 ± 0.11	0.103 ± 0.054
		**non-emitters**	5.4 ± 0.5 *	0.19 ± 0.02	0.278 ± 0.010 *	2.14 ± 0.10 *	0.080 ± 0.051
	**37**	**emitters**	8.1 ± 0.8	0.21 ± 0.02	0.392 ± 0.013	1.57 ± 0.04	0.116 ± 0.053
		**non-emitters**	5.0 ± 0.4 *	0.20 ± 0.01	0.338 ± 0.012 *	1.81 ± 0.04 *	0.085 ± 0.056
